# Satisfaction of people at post-working age with pharmacists’ health promotion in Poland

**DOI:** 10.1186/s12889-024-17751-3

**Published:** 2024-01-23

**Authors:** Dorota Raczkiewicz, Jakub Owoc, Iwona Bojar, Beata Sarecka-Hujar

**Affiliations:** 1 Department of Medical Statistics, School of Public Health, Centre of Postgraduate Medical Education, Kleczewska 61/63 Street, 01-826 Warsaw, Poland; 2 National Institute of Geriatrics, Rheumatology and Rehabilitation, Spartańska 1 Street, 02-637 Warsaw, Poland; 3 Department of Women’s Health, Institute of Rural Health in Lublin, Jaczewskiego 2 Street, 20- 090 Lublin, Poland; 4 Department of Basic Biomedical Science, Faculty of Pharmaceutical Sciences in Sosnowiec, Medical University of Silesia in Katowice, Kasztanowa 3 Street, 41-200 Sosnowiec, Poland

**Keywords:** Health promotion, Health behaviors,, Pharmacists, Community pharmacy, Post-working age

## Abstract

**Background:**

The study aimed to analyze how people at post-working age evaluate health promotion conducted for them by pharmacists in community pharmacies in Poland. We also assessed whether this evaluation is correlated with the frequency of health behaviors.

**Methods:**

The study comprised 712 Polish people at post-working age (retired), including women 60 + and men 65+. Health Behaviors Inventory and authors’ Questionnaire for Evaluation of Pharmacists’ Health Promotion were used.

**Results:**

Conducting health promotion by pharmacists in community pharmacies is relevant in the opinion of post-working-aged people (5.8 on average in the scale of 1–10). However, the patients were not satisfied with the reliability (4.7), accessibility (4.7), communicativeness (5.0), and effectiveness (4.6) of health promotion provided by pharmacists for them. The empathy and politeness of pharmacists during health promotion were rated neutrally (5.4, i.e. neither good nor bad). The evaluations of reliability, accessibility, communicativeness, empathy and politeness, relevance, and effectiveness of pharmacists’ health promotion did not correlate with age, marital status, place of residence, type of job in the past, or chronic pain currently (*p* > 0.05). The men evaluated accessibility higher than the women (5.1 vs. 4.6, *p* = 0.049), but the other domains were evaluated similarly by both genders (*p* > 0.05). All the domains of pharmacists’ health promotion were assessed the better the higher the frequency of health behaviors the post-working aged people was.

**Conclusions:**

People in post-working age assessed that health promotion conducted by pharmacists in community pharmacies is important, however they were not satisfied with the reliability, accessibility, communicativeness, and effectiveness of health promotion conducted by pharmacists.

**Supplementary Information:**

The online version contains supplementary material available at 10.1186/s12889-024-17751-3.

## Background

The aging of populations is currently one of the growing problems worldwide [1, 2]. It results from the significant extension of human life compared to the previous decades and is additionally deepened by the low fertility rate [2]. The average lifespan of a man is 70 years and a woman is 80 years. In Poland, over the last 15 years, the percentage of people aged 60 and above has increased to over 25%, from 17.2% in 2005 and 22.9% in 2015 to 25.6% in 2020. The largest age group among Polish people over 60 is people between 60 and 64 years of age (this percentage was 27.3% in 2020) [3].

Elderly age is often associated with problems and chronic diseases affecting everyday functioning [4]. The most common diseases in the post-working age include diabetes, cardiovascular diseases, cancer, balance disorders, locomotion impairment, dementia syndromes, and depression [4]. In addition, among people over 70, there is an increased number of women who are burdened with health problems related to menopause, when hormonal, biological, and clinical changes occur in their bodies [5, 6]. Currently, the average woman lives 1/3 of her life in the postmenopausal period, when the risk of developing many diseases is increased [7].

The number of new cancer cases in Poland has doubled in the last 30 years [8]. According to the Polish Ministry of Health, it is expected that in 2029 the number of malignant tumors will increase significantly compared to 2016, and the most common will be lung cancer [9]. In the case of oncological diseases, the largest increase in the number of new cases will concern people aged 75–84. An increase in morbidity is also estimated in the case of diabetes, chronic obstructive pulmonary disease, interstitial lung diseases, or diseases of the central nervous system, especially Alzheimer’s disease.

Therefore, the change in the social structure in favor of older age groups has far-reaching consequences, including social, economic, and medical ones, with an increased demand for treatment, rehabilitation, and care, which is undoubtedly a great challenge for the state health care system.

Due to the frequent visits to doctors of various specialties, elderly people also often visit pharmacies. Recently, it was demonstrated that patients visit pharmacies significantly more often than doctors [10]. This is undoubtedly related to the number of pharmacies and easy access to them, as they are usually located in city centers, supermarkets or shopping centers. Also, pharmacies are often the first and sometimes the only point of contact with health services, especially in poor communities. In many countries, pharmacists play an important role in immunization and vaccination education [11, 12]. In Poland, before the COVID-19 pandemic, pharmacists were not allowed to vaccinate patients. However, a legislative change was introduced to perform vaccinations in pharmacies, and in consequence, the number of vaccinations against COVID-19 increased [13]. Polish patients declared great satisfaction with vaccinations performed by pharmacists and also indicated a sense of safety during the procedure [13]. Polish pharmacists themselves also drew attention to the numerous advantages of vaccines in pharmacies, including improving the overall vaccination rate and an important role in promoting vaccinations [14]. Additionally, in Australian pharmacies, for example, the possibility of implementing a weight control program in overweight and obese patients was investigated [15] and in Polish pharmacies, a model of screening and counseling on blood pressure control was tested [16].

All of this puts pharmacists in a unique position to help patients, especially elderly patients, as the use of medications by them can be associated with many complications, including dosing difficulties. Therefore, such assistance to patients ultimately improves public health. The vast majority of elderly people take 3 or more medications (polypharmacy) which is associated with the intensification of possible side effects or a decrease in the quality of life [17, 18]. Earlier, the idea of pharmaceutical pictograms was verified to improve the use of drugs prescribed. Pictograms could help patients to adhere to medical advice and reduce the potential risks or errors associated with the misuse of medications. Merks et al. [19] demonstrated that pictograms significantly helped patients not to omit doses, not to crush tablets, to use a proper number of tablets per day and to use tablets at the proper time. Health behaviors contribute to the development of good habits throughout the patient’s life. The number of healthy behaviors among patients can indicate the extent to which good lifestyle habits contribute to an active and healthy life in older years. Understanding what is the impact of biopsychosocial and environmental aspects on the accumulation of health behaviors would be useful, among others, in order to create preventive programs in primary health care [20].

The aim of the present study was to analyze how people at post-working age evaluate health promotion conducted for them by pharmacists in community pharmacies in Poland. We also assessed whether this evaluation is correlated with the frequency of health behaviors.

## Methods

### The study group

The study was conducted in a group of 712 participants, including 589 women (82.72%) and 123 men (17.28%). All of them were at post-working age (retired), which in Poland is over 60 years old for women and over 65 years old for men.

The survey was conducted in community pharmacies in Poland. There were approximately 12 thousand community pharmacies in Poland listed on the National Health Fund website. Due to some financial and organizational limitations, 120 community pharmacies from this list were selected for the study, which constitutes 1% of all community pharmacies in the country. A systematic sample of community pharmacies was used, where every 100th pharmacy from the NHF list was chosen. Each selected pharmacy was sent 10 copies of the survey questionnaire with short instructions on how to fill them in and how to select a sample of participants. The selection of the sample in the pharmacies was random and systematic. The pharmacists were asked to select every tenth participant among those meeting the criteria for inclusion in the study.

The survey was anonymous, preceded by the consent of the person to participate, and it was conducted in conditions that enabled reliable and honest answers to the questions contained in the questionnaire. In each pharmacy, there was a specially designated quiet place where the patient had an opportunity to fill out the questionnaires by themselves. Had the patient needed some help with reading the questions, they could ask the pharmacists. The completed questionnaires were submitted by the participants in a designated place in the pharmacy. All collected questionnaires were sent back by the post to Institute of Rural Health in Lublin (Poland).

Of the obtained questionnaires, those that were largely incomplete and/or incorrectly completed were rejected. In total, 712 correctly completed questionnaires were included in the study. The survey response rate was 60%.

### Description of the survey questionnaire

The research tool was a questionnaire consisting of three parts:

Part I included questions regarding the demographic and social characteristics of the respondents. The respondents were asked about gender, age, level of education, place of residence, type of job in the past, chronic pain currently, and chronic disorders;

Part II– Health Behaviors Inventory;

Part III– Respondents’ Evaluation of Pharmacists’ Health Promotion.

### Health behaviors inventory

The Health Behavior Inventory by Juczyński [21] is standardized to examine both healthy and sick adults (see Supplementary file). It contains 24 items describing various health-related behaviors. A respondent indicates how often during the last year they performed given activities on a 5-point scale, where: 1 means “almost never”, 2– “rarely”, 3– “sometimes”, 4– “often”, and 5– “almost always”.

The sum of the numerical values indicated by a respondent gives a general indicator of the frequency of health behaviors. It assumes values from 24 to 120 scores. The higher the score is, the higher the frequency of the declared health behaviors. The general indicator of the frequency of health behaviors is converted into stens, and they are then converted into three ranges of frequency of health behaviors: low (24–71 scores), average (72–86), and high frequency of health behaviors (87–120). The three ranges of frequency of health behaviors were determined by Juczyński [21] and we just followed the pattern.

The frequency for the 4 subscales of health behaviors is calculated separately: good eating habits (items: 1,5,9,13,17,21), preventive behaviors (items: 2,6,10,14,18,22), positive mental attitude (items: 3,7,11,15,19,23), health practices (items: 4,8,12,16,20,24). Their scores are calculated as an arithmetic mean of scores obtained from the respondent’s answers to individual items.

### Questionnaire for evaluation of pharmacists’ health promotion

In order to develop this questionnaire we analyzed the literature regarding the subject matter as well as other questionnaires which analyze similar subject matter used worldwide. A preliminary version of the questionnaire was created. We checked if the respondents clearly understood the questions in the pitot study on the sample of 30 respondents. Some questions were modified and adjusted. The questionnaire was validated. We calculated the statistical reliability and internal consistency of the 6 complex domains. Cronbach’s α reliability coefficient and mean correlation r coefficients were calculated.

A questionnaire for evaluation of pharmacists’ health promotion was prepared by the authors and it included 6 domains: reliability, accessibility, communicativeness, empathy and politeness, relevance, and effectiveness of health promotion conducted by pharmacists on customers in community pharmacies (see Supplementary file).

Each of the above-mentioned 6 domains contained 30 items regarding knowledge about health, prevention of diseases, and help in dealing with health problems.

All the items were evaluated on a scale from 1 to 10, where 1 - very bad, and 10 - very good. The scale midpoint was 5.5, i.e. scores below 5.5 indicate bad evaluation and scores above 5.5 indicate good evaluation. The scores which did not significantly differ from 5.5 were neutral (neither good nor bad).

### Statistical methods

The data were statistically analyzed using STATISTICA 13 software. Minimum and maximum values as well as means (M) and standard deviations (SD) were estimated for numerical variables, while absolute numbers (n) and percentages (%) of the occurrence of categories for categorical variables.

Cronbach’s α reliability coefficient and mean correlation r coefficients were used to check the reliability and the internal consistency of the 6 complex domains.

The following statistical tests were used:

- Student t-test to compare numerical variables between genders;
- chi-square test to compare categorical variables between genders;
- one sample Student t-test to compare scores with scale mid-point;
- Pearson correlation coefficient r to correlate evaluations of pharmacists’ health promotion with age and the frequency of health behaviors;
- chi-square test to correlate evaluations of pharmacists’ health promotion with the level of education, marital status, place of residence, type of job in the past, or suffering from chronic pain;
- F-test analysis of variance to compare evaluations of pharmacists’ health promotion between the 3 groups of respondents: with high, moderate, and low frequency of health behaviors.

Due to the large sample size, the parameter estimators are asymptotically normally distributed due to the central limit theorem, so parametric tests were used.

The significance level was set at *p* < 0.05.

## Results

### Characteristics of the study group

The respondents in the study group were aged 60–79 years, 68.4 ± 5.2 years, on average. The surveyed women were younger, higher educated, lived in bigger cities, were more often divorced, and more often had an intellectual job in the past, than the surveyed men (Table 1). Chronic pain was declared by 32% of the respondents and its prevalence did not differ significantly between the genders.

**Table 1:** Characteristics of the respondents

Variable	IU or category	Parameter	Total (*N* = 712)	Men (*N* = 123)	Women (*N* = 589)	p ^1^ for comparison between men and women
Age	years	Min−Max, M ± SD	60−79, 68.4 ± 5.2	65−79, 70.0 ± 4.7	60−79, 68.0 ± 5.2	< 0.001
Level of education	primary	n (%)	26 (3.65)	8 (6.50)	18 (3.06)	< 0.001
	basic vocational	n (%)	70 (9.83)	26 (21.14)	44 (7.47)	
	secondary	n (%)	357 (50.14)	46 (37.40)	311 (52.80)	
	university	n (%)	259 (36.38)	43 (34.96)	216 (36.67)	
Marital status	single	n (%)	30 (4.21)	6 (4.88)	24 (4.07)	0.001
	married	n (%)	420 (58.99)	87 (70.73)	333 (56.54)	
	divorced	n (%)	252 (35.39)	26 (21.14)	226 (38.37)	
	co-habiting	n (%)	10 (1.40)	4 (3.25)	6 (1.02)	
Place of residence	rural	n (%)	76 (10.67)	20 (16.26)	56 (9.51)	< 0.001
	small town ^2^	n (%)	186 (26.12)	43 (34.96)	143 (24.28)	
	medium city ^3^	n (%)	410 (57.58)	58 (47.15)	352 (59.76)	
	city ^4^	n (%)	40 (5.62)	2 (1.63)	38 (6.45)	
Type of job in the past	intellectual	n (%)	458 (65.62)	57 (47.90)	401 (69.26)	< 0.001
	physical	n (%)	64 (9.17)	26 (21.85)	38 (6.56)	
	mixed	n (%)	176 (25.21)	36 (30.25)	140 (24.18)	
Chronic pain currently	yes	n (%)	228 (32.02)	40 (32.52)	188 (31.92)	0.896
	no	n (%)	484 (67.98)	83 (67.48)	401 (68.08)	

^1^ p for the student t-test to compare age between genders or p for the chi-square test to compare other variables between genders

^2^ up to 25 thousand inhabitants, ^3^ 25–150 thousand inhabitants, ^4^ above 150 thousand inhabitants

**Fig. 1:**
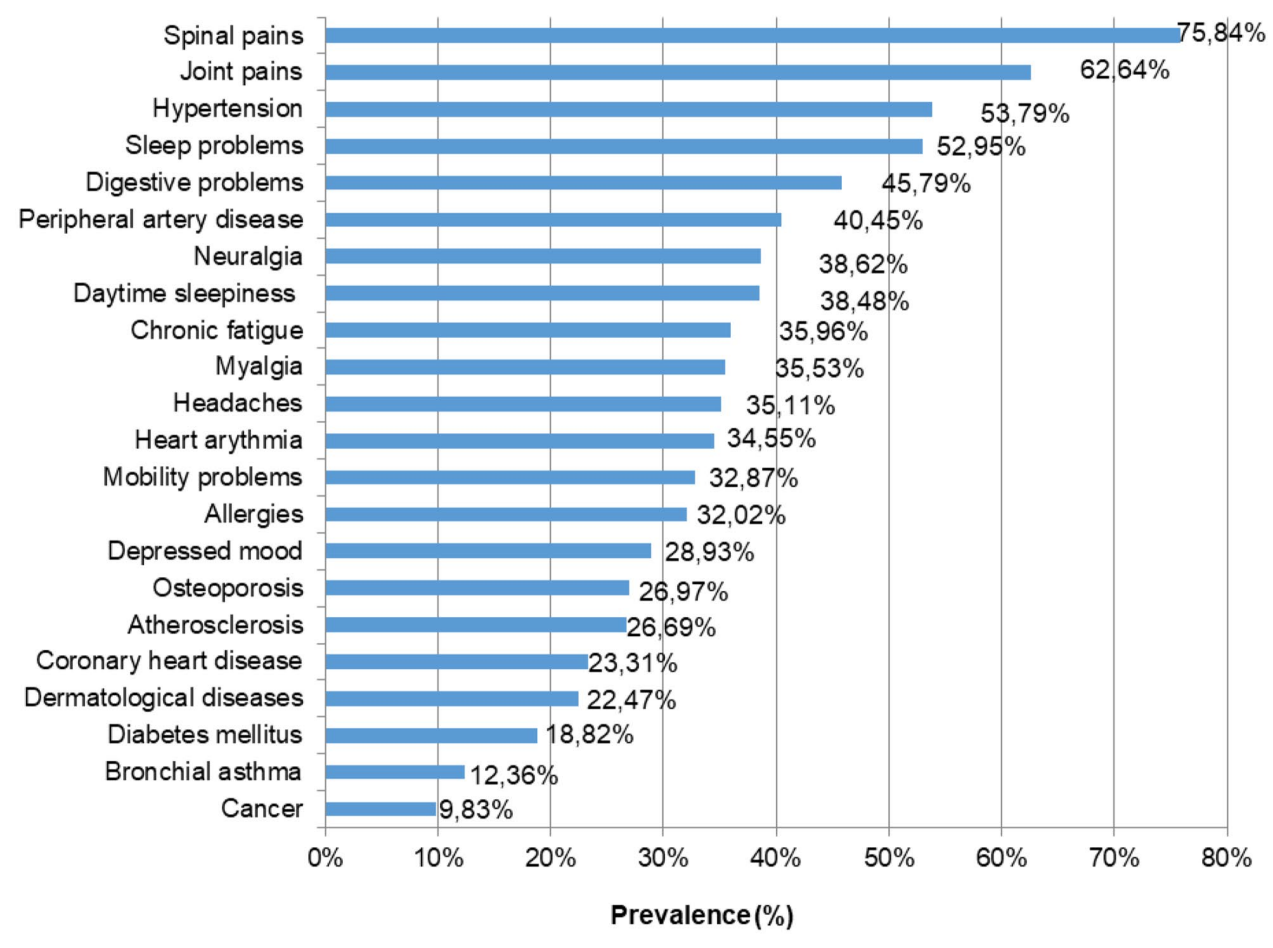
The prevalence of chronic disorders in the total sample. Results are presented as % out of the total sample (*N* = 712)

The respondents were asked about the prevalence of chronic disorders. Figure 1 presents the prevalence of chronic disorders in the total sample. Most of the respondents suffered from spinal and joint pains (76% and 63%, respectively and hypertension (54%) as well as sleep and digestive problems (53% and 46%, respectively)). The following disorders were reported by 30–40% of the respondents: peripheral artery disease, neuralgia, daytime sleepiness, chronic fatigue, myalgia, headaches, heart arrhythmia, mobility problems, allergies, 20–30% of the respondents suffered from depressed mood, osteoporosis, atherosclerosis, coronary heart and dermatological diseases., each, 19% had diabetes mellitus, 12% had bronchial asthma and 10% suffered from cancer.

**Fig. 2:**
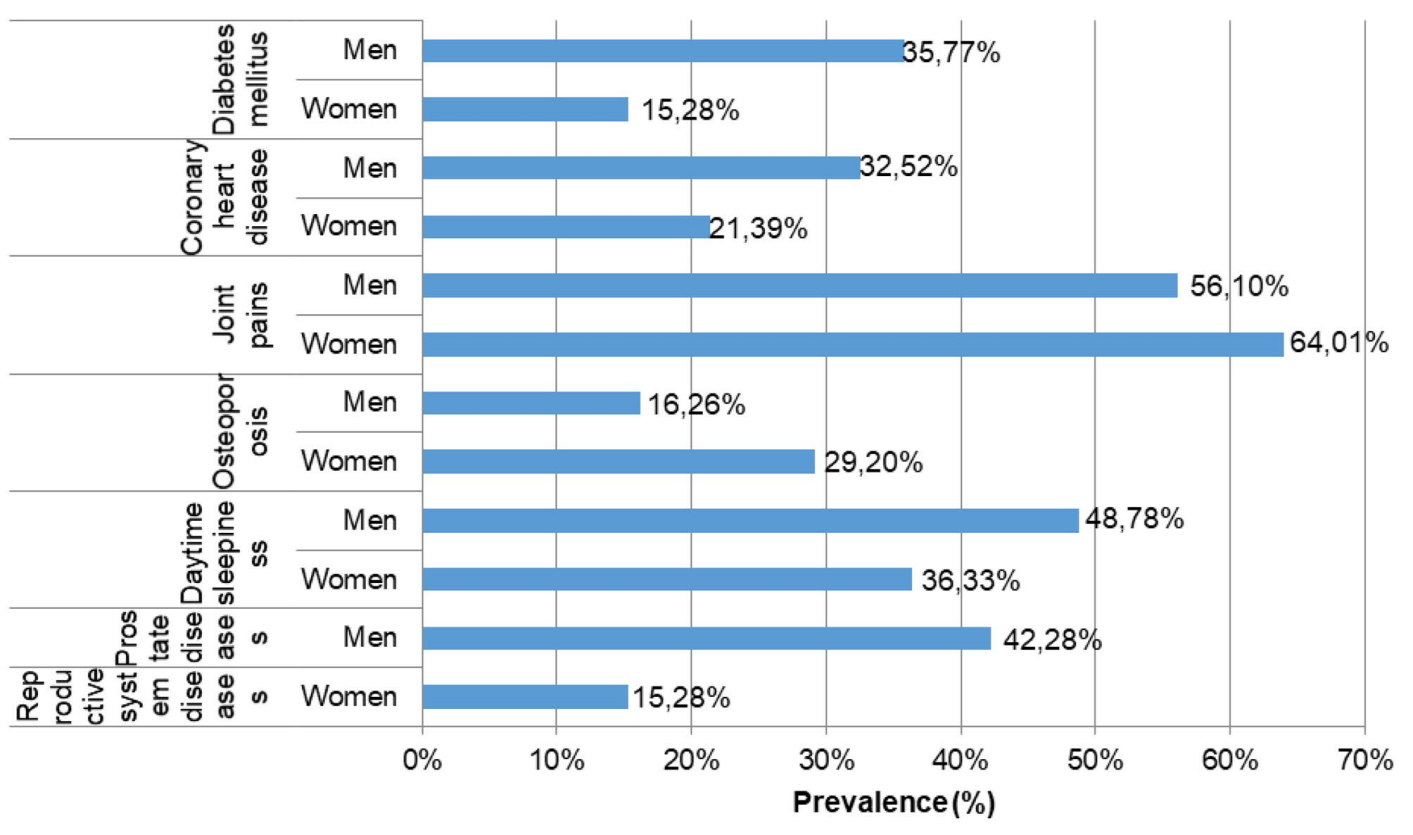
The prevalence of chronic disorders occurred with different frequencies in both genders. The results are presented as % out of men (*N* = 123) or women (*N* = 589)

Figure 2 presents those chronic disorders that occurred with different prevalence in the respondents of both genders. A higher percentage of the surveyed men suffered from diabetes mellitus (36%) than the surveyed women (15%). The same difference concerned the following diseases: coronary heart disease (33% and 21%, respectively), and daytime sleepiness (49% and 36%, respectively). However, a higher percentage of the surveyed women suffered from joint pains (64% vs. 56%) and osteoporosis (29% vs. 16%) than men. 42% of the surveyed men declared prostate diseases, while 15% of the surveyed women - reproductive system diseases.

### Frequency of health behaviors in the study group

The frequency of health behaviors in total and in the 4 subscales (good eating habits, preventive behaviors, positive mental attitude and health practices) did not differ significantly between the surveyed women and men (Table 2). The frequency of health behaviors in total was on average 88.68 ± 14.44. Almost half of the respondents had high frequency of health behaviors (49%), 32% - moderate, and 19% - low. The average scores of the 4 subscales (good eating habits, preventive behaviors, positive mental attitude, and health practices) were from 3.6 to 3.8, which means that the respondents behaved in a healthy way with a frequency between “sometimes” and “often”.

**Table 2:** Frequency of health behaviors in the respondents

Domain	Frequency	Parameter	Total (*N* = 712)	Men (*N* = 123)	Women (*N* = 589)	p ^1^ for comparison between men and women
Total	score ^2^	M ± SD	88.68 ± 14.44	87.42 ± 13.57	88.94 ± 14.62	0.294
	low	n (%)	134 (19.36)	22 (18.49)	112 (19.55)	0.890
	moderate	n (%)	220 (31.79)	40 (33.61)	180 (31.41)	
	high	n (%)	338 (48.84)	57 (47.90)	281 (49.04)	
Good eating habits	score ^3^	M ± SD	3.7 ± 0.8	3.6 ± 0.8	3.7 ± 0.8	0.262
Preventive behaviors	score ^3^	M ± SD	3.8 ± 0.7	3.7 ± 0.7	3.8 ± 0.7	0.137
Positive mental attitude	score ^3^	M ± SD	3.7 ± 0.7	3.7 ± 0.5	3.7 ± 0.7	0.732
Health practices	score ^3^	M ± SD	3.6 ± 0.7	3.6 ± 0.6	3.6 ± 0.7	0.651

^1^ p for Student t-test to compare scores between genders or p for chi-square test to compare low, moderate and high frequency of health behaviors between genders

^2^ scale 24–120

^3^ 5-point scale, where 1– almost never, 2– rarely, 3– sometimes, 4– often, 5– almost always

### The respondents’ evaluation of pharmacists’ health promotion

The statistical reliability and internal consistency of the 6 complex domains were estimated as good. Cronbach’s α and mean correlation coefficient were as follows: for reliability α = 0.990 and *r* = 0.785; for accessibility α = 0.993 and *r* = 0.835; for communicativeness α = 0.944 and *r* = 0.856; for empathy and politeness α = 0.995 and *r* = 0.870; for relevance α = 0.993 and *r* = 0.834; for effectiveness α = 0.994 and *r* = 0.849.

Among the 6 domains in which the respondents evaluated pharmacists’ health promotion (Table 3), the respondents rated the relevance of pharmacists’ health promotion as the highest and as “good” (5.8 on average and significantly above the scale mid-point). The lower scores were given to empathy and politeness of pharmacists’ health promotion (5.4 on average), but this result did not differ significantly from the scale mid-point, i.e. it was neither “good” nor “bad”. On the other hand, the following were assessed significantly “bad” by the respondents: effectiveness, reliability, accessibility, and communicativeness of pharmacists’ health promotion (significantly below the scale mid-point).

**Table 3:** Evaluation of pharmacists’ health promotion by the respondents

Domain	Total (M ± SD)	p ^1^ for comparison with scale midpoint = 5.5	Men (M ± SD)	Women (M ± SD)	p ^2^ for comparison between men and women
Reliability	4.7 ± 2.6	< 0.001	4.8 ± 2.7	4.7 ± 2.6	0.669
Accessibility	4.7 ± 2.7	< 0.001	5.1 ± 2.9	4.6 ± 2.6	0.049
Communicativeness	5.0 ± 2.8	< 0.001	5.3 ± 3.0	5.0 ± 2.8	0.179
Empathy and politeness	5.4 ± 2.9	0.368	5.6 ± 3.0	5.4 ± 2.8	0.427
Relevance	5.8 ± 2.8	0.007	5.9 ± 2.8	5.8 ± 2.7	0.615
Effectiveness	4.6 ± 2.7	< 0.001	4.8 ± 2.8	4.5 ± 2.7	0.196

10-point scale from 1– very bad to 10– very good. Scale midpoint = 5.5

^1^ p for one sample Student t-test to compare scores with scale midpoint

^2^ p for two samples Student t-test to compare scores between genders

Accessibility was the only domain that was evaluated significantly differently between the genders (*p* = 0.049). The surveyed men evaluated it higher than the surveyed women (5.1 vs. 4.6, on average). The other domains were evaluated similarly by both genders (Table 3).

Moreover, the evaluation of all 6 domains did not depend on the respondents’ age, level of education, marital status, place of residence, type of job in the past or suffering from chronic pain currently (*p* > 0.05).

### The correlations between the frequency of health behaviors and the evaluation of pharmacists’ health promotion

Significant positive linear correlations were found between the frequency of health behaviors (in total and in the 4 subscales: good eating habits, preventive behaviors, positive mental attitude, and health practices) and the evaluation of pharmacists’ health promotion (in all the 6 domains: reliability, accessibility, communicativeness, empathy, and politeness, relevance, effectiveness) (Table 4). This means that the more frequently the respondents behaved in a healthy way, the better they evaluated the pharmacists’ health promotion.

**Table 4:** Correlations between the frequency of health behaviors and the evaluation of pharmacists’ health promotion

Domain	Frequency of health behaviors									
	Good eating habits		Preventive behaviors		Positive mental attitude		Health practices		Total health behaviors	
	r	p	r	p	r	p	r	p	r	p
Reliability	0.111	0.004	0.132	0.001	0.130	0.001	0.139	< 0.000	0.154	< 0.001
Accessibility	0.143	< 0.001	0.164	< 0.001	0.166	< 0.001	0.141	< 0.000	0.185	< 0.001
Communicativeness	0.128	0.001	0.153	< 0.001	0.177	< 0.001	0.149	< 0.000	0.182	< 0.001
Empathy and politeness	0.190	< 0.001	0.188	< 0.001	0.190	< 0.001	0.159	< 0.000	0.220	< 0.001
Relevance	0.125	0.001	0.185	< 0.001	0.177	< 0.001	0.090	0.021	0.173	< 0.001
Effectiveness	0.153	< 0.001	0.184	< 0.001	0.175	< 0.001	0.133	0.001	0.194	< 0.001

r– Pearson correlation coefficient

**Fig. 3:**
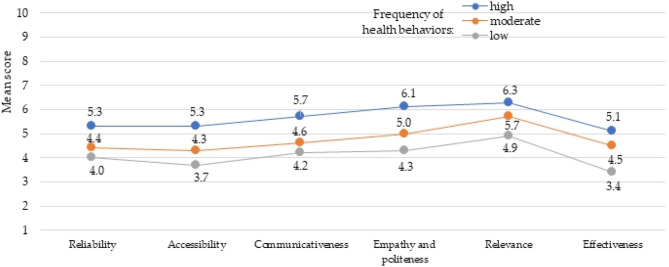
Evaluation of pharmacists’ health promotion versus frequency of health behaviors. *p* < 0.001 for each domain. 10-point scale from 1– very bad to 10– very good

A coherent conclusion was obtained when comparing the respondents’ evaluation of the pharmacists’ health promotion, between the three groups of respondents: with high, moderate and low frequency of health behaviors in total (Fig. 3). The respondents with a high frequency of health behaviors rated all the scales of the pharmacists’ health promotion the highest. The respondents with a low frequency of health behaviors rated all the scales of the pharmacists’ health promotion the lowest. The respondents with a moderate frequency of health behaviors rated all the scales of the pharmacists’ health promotion at medium level.

## Discussion

In our study, the vast majority of post-working age respondents were women. In Poland, statistically more women than men look after their and their family’s health. This has cultural and historical reasons therefore women more often visit pharmacies. The numbers of women and men in our study reflect the natural situation in the country.

Health-promoting activities are particularly important for patients aged 60 and over, due to the multimorbidity which is related to elderly age [22]. Among the respondents, the largest percentage (over 50%) suffer from hypertension and sleep problems. Scientific data indicate that the prevalence of hypertension in the group of people aged 65–94 years is over 70% [23]. However, due to the projected increase in the number of elderly people over 65 in the population, it is estimated that the prevalence of hypertension will also increase [24]. Other chronic diseases suffered by the respondents include digestive disorders, peripheral artery disease, mobility problems, allergies, headaches, muscle pains, chronic fatigue, atherosclerosis, depressed mood, dermatological diseases and diabetes. Some gender-related differences in the incidence of individual diseases were found. Coronary heart disease as well as daytime sleepiness were more common in the men than in the women surveyed (33% vs. 22%, respectively, and 49% vs. 36%, respectively). There was also a higher prevalence of diabetes in men than in women (36% vs. 15%, respectively). In the study by Nordström et al. [25], the incidence of type 2 diabetes in the group of patients over 70 years of age also differed significantly between genders (14.6% in men vs. 9.1% in women). On the other hand, in the women surveyed in this study, joint pain and osteoporosis were more common. Prostate diseases were declared by 42 of the surveyed men and 15% of the women had diseases of the reproductive organs.

The simultaneous occurrence of many diseases affecting various organs of the body in the elderly necessitates the provision of appropriate care by the entire health service to this social group. Although in the study by Toner et al. [26], the old age itself was not recognized as an obstacle in conducting pharmaceutical counseling, the multimorbidity was. The appropriate intervention of a pharmacist, as a health care representative with the greatest knowledge about drugs, helps in the proper conduct of pharmacotherapy and obtaining its proper effects, but also in preventing the occurrence of side effects of medications.

In the present study, almost half of the respondents had a high frequency of health behaviors (49%). The respondents behaved in a healthy way with a frequency between “sometimes” and “often”. The frequency of health behaviors in the total score and within the subscales of good eating habits, preventive behaviors, positive mental attitude, and health practices did not differ significantly between the women and the men.

Health education increases people’s awareness in the field of pro-health behaviors [27]. The knowledge on how to change a harmful lifestyle and make decisions conducive to their own health is especially important for elderly patients. Thanks to this, it is possible to create health attitudes in society, which in turn can be used in various preventive campaigns. The availability of health education in general and the availability of disease prevention by pharmacists for patients were evaluated significantly better by the surveyed men than by the women. In the remaining scales, the assessment of pharmacists in the field of health promotion did not depend on the gender of the respondents. There was also no correlation between the age of the respondents, place of residence, the nature of the work performed in the past by the respondents, and the assessment of pharmacists in the field of health promotion.

In the present study, the respondents with a high frequency of health behaviors rated pharmacists the best in terms of health promotion, in contrast to the respondents with an average overall intensity of health behaviors and low overall intensity of health-related behaviors. The effectiveness, reliability, and accessibility of health education provided by pharmacists to patients were significantly better assessed by those patients, according to whom their health was “good” than by those who assessed their health as “bad” or “decent”.

An earlier research on a group of 252 patients and 620 pharmacists conducted in South Korea showed that almost 50% of pharmacists and 34% of patients were satisfied with the pharmaceutical counseling provided. The main reason for dissatisfaction with pharmacists’ advice both for the patients and the pharmacists themselves was, insufficient time spent on counseling (51%) [28]. In patient care provided in a pharmacy, an important aspect is a satisfaction and sense of being taken care of by patients. In 2012, the results of research conducted in Poland on the level of patient satisfaction with services provided in pharmacies were published [29]. In this study, also women predominated, but the age of the respondents was significantly lower than in our study (20–29 years). It has been shown that the majority of people participating in the study would be interested in additional activities for patients carried out in pharmacies, e.g. blood glucose and cholesterol measurement, blood pressure or weight measurement. In addition, almost half of the respondents admitted that they would benefit from training conducted by pharmacists, e.g. in the use of an inhaler or advice on improving the quality of life (diet, physical activity, fighting addictions). On the other hand, in terms of advice on the therapy prescribed by the doctor, 16% of the respondents in the study definitely did not think that the pharmacist clearly explained the dosage of drugs during the prescription, 14% of people did not know the side effects of the drugs used, and according to 11% - the pharmacist did not explain how to dose the prescribed medications [29].

The respondents who believed that pharmacists should conduct health education in pharmacies rated pharmacists significantly better in terms of health promotion than those who believed that pharmacists should not do it or had no opinion on this subject. In a cross-sectional study of 287 patients served in 5 pharmacies of the University Hospital in Gondar (Ethiopia), slightly more than half claimed that they were satisfied with the services received at the pharmacy [30]. However, the level of patient satisfaction was correlated with the type of pharmacy where they were served– the ophthalmology pharmacy was rated the highest. On the other hand, the patients’ expectations of pharmaceutical services were correlated with their monthly income– people with lower incomes had lower expectations than those with higher incomes.

Many studies have demonstrated a high level of satisfaction with certain aspects of the services provided in pharmacies, such as the way pharmacists work, providing instructions on drug dosing or the possibility of clarifying doubts [31, 32]. However, there is also data indicating a low level of patient satisfaction with the way they were served by the pharmacists, e.g. due to poor understanding of the content [33]. This study showed a neutral (neither positive nor negative) assessment of the sensitivity and kindness of pharmacists towards patients during health education. On the other hand, the following were assessed significantly negatively by the respondents: effectiveness, reliability, accessibility, and communicativeness of health education conducted for patients by pharmacists. Communication barriers between pharmacists and patients in providing health information were also reported in the previous studies [34].

Surprising results in terms of communication between the pharmacist and the patient were reported by Swedish research [35]. The authors demonstrated that during patients’ visits to pharmacies in Sweden, there was almost no dialogue regarding medical issues, while 40% of the conversations concerned non-medical issues, and almost half of the meetings were silent [35]. Advising patients on their prescribed medications is a priority for the pharmacist to improve treatment outcomes, increase compliance, and increase patient safety. The lack of a counseling conversation may therefore result in little benefit that the patient will derive from the treatment. On the other hand, it was observed that Danish pharmacists initiate the majority (60%) of conversations with patients, of which a large part (38%) is not undertaken by them, while patients started conversations with the pharmacist only in 13% of cases [36]. The authors also showed that patients’ interest in talking to a pharmacist depends on their age and gender. In turn, in the study by Gammie et al. [37], who conducted simultaneous surveys among the public (street survey) and among pharmacists, the majority of the respondents said that they prefer not to interrupt a pharmacist who is busy in the pharmacy. Thus, the lack of effective communication between the patient and the pharmacist may result from such a belief.

In the previous study where pharmacists assessed themselves [38], the married pharmacists assessed significantly better health education provided by them in the following domains: communicativeness regarding dealing with health problems, sensitivity and kindness regarding health knowledge.

Therefore, various factors, including social and cultural, should be taken into account by pharmacists to optimize communication with the patient when purchasing drugs. In turn, in our study, the availability of health education in general and its availability in the field of disease prevention conducted by pharmacists, as well as the communicativeness of health education conducted by pharmacists for the patient with regards to help in dealing with health problems, were assessed significantly better by the respondents with primary education than other respondents. Our previous studies analyzed how pharmacy staff and pharmacy students in Poland evaluated their own qualifications, competencies, relevance, motivation, and effectiveness of health promotion [38, 39]. The relevance of health promotion was rated by the pharmacy staff at a level of 7.0 ± 1.7, in the pharmacy students the relevance was rated at a higher level of 7.4 ± 1.8 while in the present study in patients at post-working age, the relevance was rated at a lower level of 5.8 ± 2.8. Similarly, the effectiveness of health promotion was rated at the highest level by pharmacy students (6.4 ± 1.8) compared to pharmacy staff (5.7 ± 1.9) and people at post-working age (4.6 ± 2.7) [38, 39].

Data on the perception of the role of pharmacists in the field of public health and the provision of services in the field of education and preventive health by themselves as well as by patients are necessary for the development of public health programs in public pharmacies. However, to date, few comprehensive studies on this topic have been conducted in Poland. Undoubtedly, patients’ opinions are very important in this matter because they can help shape the provision of health-promoting services in pharmacies as well as their standardization.

The limitation of the study was the analysis of only post-working age people as due to their age and multimorbidity, they more often use pharmacies and pharmaceutical care. We did not analyze other age groups, thus we were not able to perform comparisons between age subgroups.

## Conclusions

Conducting health promotion by pharmacists in community pharmacies is relevant in the opinion of post-working-aged people although they were not satisfied with the reliability, accessibility, communicativeness, and effectiveness of health promotion provided by the pharmacists for them. The empathy and politeness of pharmacists during health promotion were rated neutrally (neither good nor bad) by people at post-working age.

The evaluations of reliability, accessibility, communicativeness, empathy and politeness, relevance, and effectiveness of pharmacists’ health promotion did not correlate to age, marital status, place of residence, type of job in the past, or chronic pain currently of people at post-working age. Men evaluated accessibility higher than women, but other domains were evaluated similarly by both genders. All the domains of pharmacists’ health promotion were assessed the better, the higher the frequency of health behaviors the post-working age people had.

The role of the pharmacist in health promotion can be significant. Therefore, it is necessary to create conditions for the development of the professional independence of pharmacists and the promotion of pharmaceutical care as a set of new pharmaceutical services available to a wide range of patients. In Poland, it is important to systematize activities aimed at a fuller use of the pharmacist’s potential in promoting projects in the area of public health, including the promotion of pro-health behaviors and primary and secondary prevention of diseases.

### Electronic supplementary material

Below is the link to the electronic supplementary material.

Supplementary Material 1

Supplementary Material 2

## Data Availability

The data presented in this study are available on request from the Corresponding Author. The data are not publicly available due to privacy restrictions.

## Abbreviations

M: Mean
SD: Standard deviations
N: Absolute numbers
