# Supplementary material for: Satisfaction of people at post-working age with pharmacists’ health promotion in Poland
Source: BMC Public Health. 2024 Jan 23;24:281. doi: 10.1186/s12889-024-17751-3 (PMC10807121; doi:10.1186/s12889-024-17751-3)
Supplement: Supplementary file 1 — Supplementary Material 1 [file 12889_2024_17751_MOESM1_ESM.docx]

**The questionnaire**

**of reliability, accessibility, communicativeness, empathy and politeness, relevance, and effectiveness of health promotion conducted by pharmacists**

**for customers in community pharmacies.**

Please put **X** to assess every item in a 10-point scale:

1 – very low, 10 – very high.

****************************************************************

1. **Reliability**

How do you assess reliability of health promotion in a pharmacy in the following items?

|  | **Health knowledge** | **1** | **2** | **3** | **4** | **5** | **6** | **7** | **8** | **9** | **10** |
| --- | --- | --- | --- | --- | --- | --- | --- | --- | --- | --- | --- |
| 1. | Individual and social costs of health, disease and disability |  |  |  |  |  |  |  |  |  |  |
| 2. | Healthy nutrition as a precondition for building health potential |  |  |  |  |  |  |  |  |  |  |
| 3. | Physical activity and mobility as a source of health |  |  |  |  |  |  |  |  |  |  |
| 4. | Classification of factors of positive and negative effect on the human body |  |  |  |  |  |  |  |  |  |  |
| 5. | Pro-health, risky and anti-health behaviors |  |  |  |  |  |  |  |  |  |  |
| 6. | Creation of safe conditions for life, work, education, leisure and communication |  |  |  |  |  |  |  |  |  |  |
| 7. | Observation of functioning of own body – scope and importance of self-examination |  |  |  |  |  |  |  |  |  |  |
| 8. | Prophylactic examinations as an element of early detection of diseases |  |  |  |  |  |  |  |  |  |  |
| 9. | Preventive vaccination – myths and reality |  |  |  |  |  |  |  |  |  |  |
| 10. | Health situations acc. to need for medical consultation and self-treatment after consultation with a pharmacist |  |  |  |  |  |  |  |  |  |  |
| 11. | Provision of pre-medical care in emergency situations |  |  |  |  |  |  |  |  |  |  |

|  | | | | | | | | | | | |
| --- | --- | --- | --- | --- | --- | --- | --- | --- | --- | --- | --- |
|  | **Prevention of** | **1** | **2** | **3** | **4** | **5** | **6** | **7** | **8** | **9** | **10** |
| 12. | cardiovascular system diseases |  |  |  |  |  |  |  |  |  |  |
| 13. | neoplasms |  |  |  |  |  |  |  |  |  |  |
| 14. | psychiatric diseases |  |  |  |  |  |  |  |  |  |  |
| 15. | respiratory system diseases |  |  |  |  |  |  |  |  |  |  |
| 16. | genitourinary system diseases |  |  |  |  |  |  |  |  |  |  |
| 17. | spine and motor system diseases |  |  |  |  |  |  |  |  |  |  |
| 18. | stomatognathic system diseases |  |  |  |  |  |  |  |  |  |  |
| 19. | metabolic diseases |  |  |  |  |  |  |  |  |  |  |
| 20. | digestive system diseases |  |  |  |  |  |  |  |  |  |  |
|  | | | | | | | | | | | |

|  | **Copying with** | **1** | **2** | **3** | **4** | **5** | **6** | **7** | **8** | **9** | **10** |
| --- | --- | --- | --- | --- | --- | --- | --- | --- | --- | --- | --- |
| 21. | stress |  |  |  |  |  |  |  |  |  |  |
| 22. | pain |  |  |  |  |  |  |  |  |  |  |
| 23. | cold, infection and temperature fluctuations |  |  |  |  |  |  |  |  |  |  |
| 24. | digestive system disorders/symptoms and food poisoning |  |  |  |  |  |  |  |  |  |  |
| 25. | skin, mucous membrane and nail disorders |  |  |  |  |  |  |  |  |  |  |
| 26. | sleeping disorders |  |  |  |  |  |  |  |  |  |  |
| 27. | occupational health and safety |  |  |  |  |  |  |  |  |  |  |
| 28. | addictions |  |  |  |  |  |  |  |  |  |  |
| 29. | oedema |  |  |  |  |  |  |  |  |  |  |
| 30. | weight disorders |  |  |  |  |  |  |  |  |  |  |
| 31. | ocular and hearing problems |  |  |  |  |  |  |  |  |  |  |
| 32. | tinea infections |  |  |  |  |  |  |  |  |  |  |
| 33. | parasite infections |  |  |  |  |  |  |  |  |  |  |
| 34 | genitourinary tract infections |  |  |  |  |  |  |  |  |  |  |
| 35. | drug and other toxins poisoning |  |  |  |  |  |  |  |  |  |  |
| 36. | allergy |  |  |  |  |  |  |  |  |  |  |

1. **Accessibility**

How do you assess accessibility of health promotion in a pharmacy in the following items?

|  | **Health knowledge** | **1** | **2** | **3** | **4** | **5** | **6** | **7** | **8** | **9** | **10** |
| --- | --- | --- | --- | --- | --- | --- | --- | --- | --- | --- | --- |
| 1. | Individual and social costs of health, disease and disability |  |  |  |  |  |  |  |  |  |  |
| 2. | Healthy nutrition as a precondition for building health potential |  |  |  |  |  |  |  |  |  |  |
| 3. | Physical activity and mobility as a source of health |  |  |  |  |  |  |  |  |  |  |
| 4. | Classification of factors of positive and negative effect on the human body |  |  |  |  |  |  |  |  |  |  |
| 5. | Pro-health, risky and anti-health behaviors |  |  |  |  |  |  |  |  |  |  |
| 6. | Creation of safe conditions for life, work, education, leisure and communication |  |  |  |  |  |  |  |  |  |  |
| 7. | Observation of functioning of own body – scope and importance of self-examination |  |  |  |  |  |  |  |  |  |  |
| 8. | Prophylactic examinations as an element of early detection of diseases |  |  |  |  |  |  |  |  |  |  |
| 9. | Preventive vaccination – myths and reality |  |  |  |  |  |  |  |  |  |  |
| 10. | Health situations acc. to need for medical consultation and self-treatment after consultation with a pharmacist |  |  |  |  |  |  |  |  |  |  |
| 11. | Provision of pre-medical care in emergency situations |  |  |  |  |  |  |  |  |  |  |

|  | | | | | | | | | | | |
| --- | --- | --- | --- | --- | --- | --- | --- | --- | --- | --- | --- |
|  | **Prevention of** | **1** | **2** | **3** | **4** | **5** | **6** | **7** | **8** | **9** | **10** |
| 12. | cardiovascular system diseases |  |  |  |  |  |  |  |  |  |  |
| 13. | neoplasms |  |  |  |  |  |  |  |  |  |  |
| 14. | psychiatric diseases |  |  |  |  |  |  |  |  |  |  |
| 15. | respiratory system diseases |  |  |  |  |  |  |  |  |  |  |
| 16. | genitourinary system diseases |  |  |  |  |  |  |  |  |  |  |
| 17. | spine and motor system diseases |  |  |  |  |  |  |  |  |  |  |
| 18. | stomatognathic system diseases |  |  |  |  |  |  |  |  |  |  |
| 19. | metabolic diseases |  |  |  |  |  |  |  |  |  |  |
| 20. | digestive system diseases |  |  |  |  |  |  |  |  |  |  |
|  | | | | | | | | | | | |

|  | **Copying with** | **1** | **2** | **3** | **4** | **5** | **6** | **7** | **8** | **9** | **10** |
| --- | --- | --- | --- | --- | --- | --- | --- | --- | --- | --- | --- |
| 21. | stress |  |  |  |  |  |  |  |  |  |  |
| 22. | pain |  |  |  |  |  |  |  |  |  |  |
| 23. | cold, infection and temperature fluctuations |  |  |  |  |  |  |  |  |  |  |
| 24. | digestive system disorders/symptoms and food poisoning |  |  |  |  |  |  |  |  |  |  |
| 25. | skin, mucous membrane and nail disorders |  |  |  |  |  |  |  |  |  |  |
| 26. | sleeping disorders |  |  |  |  |  |  |  |  |  |  |
| 27. | occupational health and safety |  |  |  |  |  |  |  |  |  |  |
| 28. | addictions |  |  |  |  |  |  |  |  |  |  |
| 29. | oedema |  |  |  |  |  |  |  |  |  |  |
| 30. | weight disorders |  |  |  |  |  |  |  |  |  |  |
| 31. | ocular and hearing problems |  |  |  |  |  |  |  |  |  |  |
| 32. | tinea infections |  |  |  |  |  |  |  |  |  |  |
| 33. | parasite infections |  |  |  |  |  |  |  |  |  |  |
| 34 | genitourinary tract infections |  |  |  |  |  |  |  |  |  |  |
| 35. | drug and other toxins poisoning |  |  |  |  |  |  |  |  |  |  |
| 36. | allergy |  |  |  |  |  |  |  |  |  |  |

1. **Communicativeness**

How do you assess communicativeness of health promotion in a pharmacy in the following items?

|  | **Health knowledge** | **1** | **2** | **3** | **4** | **5** | **6** | **7** | **8** | **9** | **10** |
| --- | --- | --- | --- | --- | --- | --- | --- | --- | --- | --- | --- |
| 1. | Individual and social costs of health, disease and disability |  |  |  |  |  |  |  |  |  |  |
| 2. | Healthy nutrition as a precondition for building health potential |  |  |  |  |  |  |  |  |  |  |
| 3. | Physical activity and mobility as a source of health |  |  |  |  |  |  |  |  |  |  |
| 4. | Classification of factors of positive and negative effect on the human body |  |  |  |  |  |  |  |  |  |  |
| 5. | Pro-health, risky and anti-health behaviors |  |  |  |  |  |  |  |  |  |  |
| 6. | Creation of safe conditions for life, work, education, leisure and communication |  |  |  |  |  |  |  |  |  |  |
| 7. | Observation of functioning of own body – scope and importance of self-examination |  |  |  |  |  |  |  |  |  |  |
| 8. | Prophylactic examinations as an element of early detection of diseases |  |  |  |  |  |  |  |  |  |  |
| 9. | Preventive vaccination – myths and reality |  |  |  |  |  |  |  |  |  |  |
| 10. | Health situations acc. to need for medical consultation and self-treatment after consultation with a pharmacist |  |  |  |  |  |  |  |  |  |  |
| 11. | Provision of pre-medical care in emergency situations |  |  |  |  |  |  |  |  |  |  |

|  | | | | | | | | | | | |
| --- | --- | --- | --- | --- | --- | --- | --- | --- | --- | --- | --- |
|  | **Prevention of** | **1** | **2** | **3** | **4** | **5** | **6** | **7** | **8** | **9** | **10** |
| 12. | cardiovascular system diseases |  |  |  |  |  |  |  |  |  |  |
| 13. | neoplasms |  |  |  |  |  |  |  |  |  |  |
| 14. | psychiatric diseases |  |  |  |  |  |  |  |  |  |  |
| 15. | respiratory system diseases |  |  |  |  |  |  |  |  |  |  |
| 16. | genitourinary system diseases |  |  |  |  |  |  |  |  |  |  |
| 17. | spine and motor system diseases |  |  |  |  |  |  |  |  |  |  |
| 18. | stomatognatic system diseases |  |  |  |  |  |  |  |  |  |  |
| 19. | metabolic diseases |  |  |  |  |  |  |  |  |  |  |
| 20. | digestive system diseases |  |  |  |  |  |  |  |  |  |  |
|  | | | | | | | | | | | |

|  | **Copying with** | **1** | **2** | **3** | **4** | **5** | **6** | **7** | **8** | **9** | **10** |
| --- | --- | --- | --- | --- | --- | --- | --- | --- | --- | --- | --- |
| 21. | stress |  |  |  |  |  |  |  |  |  |  |
| 22. | pain |  |  |  |  |  |  |  |  |  |  |
| 23. | cold, infection and temperature fluctuations |  |  |  |  |  |  |  |  |  |  |
| 24. | digestive system disorders/symptoms and food poisoning |  |  |  |  |  |  |  |  |  |  |
| 25. | skin, mucous membrane and nail disorders |  |  |  |  |  |  |  |  |  |  |
| 26. | sleeping disorders |  |  |  |  |  |  |  |  |  |  |
| 27. | occupational health and safety |  |  |  |  |  |  |  |  |  |  |
| 28. | addictions |  |  |  |  |  |  |  |  |  |  |
| 29. | oedema |  |  |  |  |  |  |  |  |  |  |
| 30. | weight disorders |  |  |  |  |  |  |  |  |  |  |
| 31. | ocular and hearing problems |  |  |  |  |  |  |  |  |  |  |
| 32. | tinea infections |  |  |  |  |  |  |  |  |  |  |
| 33. | parasite infections |  |  |  |  |  |  |  |  |  |  |
| 34 | genitourinary tract infections |  |  |  |  |  |  |  |  |  |  |
| 35. | drug and other toxins poisoning |  |  |  |  |  |  |  |  |  |  |
| 36. | allergy |  |  |  |  |  |  |  |  |  |  |

1. **Empathy and politeness**

How do you assess empathy and politeness of health promotion in a pharmacy in the following items?

|  | **Health knowledge** | **1** | **2** | **3** | **4** | **5** | **6** | **7** | **8** | **9** | **10** |
| --- | --- | --- | --- | --- | --- | --- | --- | --- | --- | --- | --- |
| 1. | Individual and social costs of health, disease and disability |  |  |  |  |  |  |  |  |  |  |
| 2. | Healthy nutrition as a precondition for building health potential |  |  |  |  |  |  |  |  |  |  |
| 3. | Physical activity and mobility as a source of health |  |  |  |  |  |  |  |  |  |  |
| 4. | Classification of factors of positive and negative effect on the human body |  |  |  |  |  |  |  |  |  |  |
| 5. | Pro-health, risky and anti-health behaviors |  |  |  |  |  |  |  |  |  |  |
| 6. | Creation of safe conditions for life, work, education, leisure and communication |  |  |  |  |  |  |  |  |  |  |
| 7. | Observation of functioning of own body – scope and importance of self-examination |  |  |  |  |  |  |  |  |  |  |
| 8. | Prophylactic examinations as an element of early detection of diseases |  |  |  |  |  |  |  |  |  |  |
| 9. | Preventive vaccination – myths and reality |  |  |  |  |  |  |  |  |  |  |
| 10. | Health situations acc. to need for medical consultation and self-treatment after consultation with a pharmacist |  |  |  |  |  |  |  |  |  |  |
| 11. | Provision of pre-medical care in emergency situations |  |  |  |  |  |  |  |  |  |  |

|  | | | | | | | | | | | |
| --- | --- | --- | --- | --- | --- | --- | --- | --- | --- | --- | --- |
|  | **Prevention of** | **1** | **2** | **3** | **4** | **5** | **6** | **7** | **8** | **9** | **10** |
| 12. | cardiovascular system diseases |  |  |  |  |  |  |  |  |  |  |
| 13. | neoplasms |  |  |  |  |  |  |  |  |  |  |
| 14. | psychiatric diseases |  |  |  |  |  |  |  |  |  |  |
| 15. | respiratory system diseases |  |  |  |  |  |  |  |  |  |  |
| 16. | genitourinary system diseases |  |  |  |  |  |  |  |  |  |  |
| 17. | spine and motor system diseases |  |  |  |  |  |  |  |  |  |  |
| 18. | stomatognathic system diseases |  |  |  |  |  |  |  |  |  |  |
| 19. | metabolic diseases |  |  |  |  |  |  |  |  |  |  |
| 20. | digestive system diseases |  |  |  |  |  |  |  |  |  |  |
|  | | | | | | | | | | | |

|  | **Copying with** | **1** | **2** | **3** | **4** | **5** | **6** | **7** | **8** | **9** | **10** |
| --- | --- | --- | --- | --- | --- | --- | --- | --- | --- | --- | --- |
| 21. | stress |  |  |  |  |  |  |  |  |  |  |
| 22. | pain |  |  |  |  |  |  |  |  |  |  |
| 23. | cold, infection and temperature fluctuations |  |  |  |  |  |  |  |  |  |  |
| 24. | digestive system disorders/symptoms and food poisoning |  |  |  |  |  |  |  |  |  |  |
| 25. | skin, mucous membrane and nail disorders |  |  |  |  |  |  |  |  |  |  |
| 26. | sleeping disorders |  |  |  |  |  |  |  |  |  |  |
| 27. | occupational health and safety |  |  |  |  |  |  |  |  |  |  |
| 28. | addictions |  |  |  |  |  |  |  |  |  |  |
| 29. | oedema |  |  |  |  |  |  |  |  |  |  |
| 30. | weight disorders |  |  |  |  |  |  |  |  |  |  |
| 31. | ocular and hearing problems |  |  |  |  |  |  |  |  |  |  |
| 32. | tinea infections |  |  |  |  |  |  |  |  |  |  |
| 33. | parasite infections |  |  |  |  |  |  |  |  |  |  |
| 34 | genitourinary tract infections |  |  |  |  |  |  |  |  |  |  |
| 35. | drug and other toxins poisoning |  |  |  |  |  |  |  |  |  |  |
| 36. | allergy |  |  |  |  |  |  |  |  |  |  |

1. **Relevance**

How do you assess relevance of health promotion in a pharmacy in the following items?

|  | **Health knowledge** | **1** | **2** | **3** | **4** | **5** | **6** | **7** | **8** | **9** | **10** |
| --- | --- | --- | --- | --- | --- | --- | --- | --- | --- | --- | --- |
| 1. | Individual and social costs of health, disease and disability |  |  |  |  |  |  |  |  |  |  |
| 2. | Healthy nutrition as a precondition for building health potential |  |  |  |  |  |  |  |  |  |  |
| 3. | Physical activity and mobility as a source of health |  |  |  |  |  |  |  |  |  |  |
| 4. | Classification of factors of positive and negative effect on the human body |  |  |  |  |  |  |  |  |  |  |
| 5. | Pro-health, risky and anti-health behaviors |  |  |  |  |  |  |  |  |  |  |
| 6. | Creation of safe conditions for life, work, education, leisure and communication |  |  |  |  |  |  |  |  |  |  |
| 7. | Observation of functioning of own body – scope and importance of self-examination |  |  |  |  |  |  |  |  |  |  |
| 8. | Prophylactic examinations as an element of early detection of diseases |  |  |  |  |  |  |  |  |  |  |
| 9. | Preventive vaccination – myths and reality |  |  |  |  |  |  |  |  |  |  |
| 10. | Health situations acc. to need for medical consultation and self-treatment after consultation with a pharmacist |  |  |  |  |  |  |  |  |  |  |
| 11. | Provision of pre-medical care in emergency situations |  |  |  |  |  |  |  |  |  |  |

|  | | | | | | | | | | | |
| --- | --- | --- | --- | --- | --- | --- | --- | --- | --- | --- | --- |
|  | **Prevention of** | **1** | **2** | **3** | **4** | **5** | **6** | **7** | **8** | **9** | **10** |
| 12. | cardiovascular system diseases |  |  |  |  |  |  |  |  |  |  |
| 13. | neoplasms |  |  |  |  |  |  |  |  |  |  |
| 14. | psychiatric diseases |  |  |  |  |  |  |  |  |  |  |
| 15. | respiratory system diseases |  |  |  |  |  |  |  |  |  |  |
| 16. | genitourinary system diseases |  |  |  |  |  |  |  |  |  |  |
| 17. | spine and motor system diseases |  |  |  |  |  |  |  |  |  |  |
| 18. | stomatognathic system diseases |  |  |  |  |  |  |  |  |  |  |
| 19. | metabolic diseases |  |  |  |  |  |  |  |  |  |  |
| 20. | digestive system diseases |  |  |  |  |  |  |  |  |  |  |
|  | | | | | | | | | | | |

|  | **Copying with** | **1** | **2** | **3** | **4** | **5** | **6** | **7** | **8** | **9** | **10** |
| --- | --- | --- | --- | --- | --- | --- | --- | --- | --- | --- | --- |
| 21. | stress |  |  |  |  |  |  |  |  |  |  |
| 22. | pain |  |  |  |  |  |  |  |  |  |  |
| 23. | cold, infection and temperature fluctuations |  |  |  |  |  |  |  |  |  |  |
| 24. | digestive system disorders/symptoms and food poisoning |  |  |  |  |  |  |  |  |  |  |
| 25. | skin, mucous membrane and nail disorders |  |  |  |  |  |  |  |  |  |  |
| 26. | sleeping disorders |  |  |  |  |  |  |  |  |  |  |
| 27. | occupational health and safety |  |  |  |  |  |  |  |  |  |  |
| 28. | addictions |  |  |  |  |  |  |  |  |  |  |
| 29. | oedema |  |  |  |  |  |  |  |  |  |  |
| 30. | weight disorders |  |  |  |  |  |  |  |  |  |  |
| 31. | ocular and hearing problems |  |  |  |  |  |  |  |  |  |  |
| 32. | tinea infections |  |  |  |  |  |  |  |  |  |  |
| 33. | parasite infections |  |  |  |  |  |  |  |  |  |  |
| 34 | genitourinary tract infections |  |  |  |  |  |  |  |  |  |  |
| 35. | drug and other toxins poisoning |  |  |  |  |  |  |  |  |  |  |
| 36. | allergy |  |  |  |  |  |  |  |  |  |  |

1. **Effectiveness**

How do you assess effectiveness in health promotion in a pharmacy in the following items?

|  | **Health knowledge** | **1** | **2** | **3** | **4** | **5** | **6** | **7** | **8** | **9** | **10** |
| --- | --- | --- | --- | --- | --- | --- | --- | --- | --- | --- | --- |
| 1. | Individual and social costs of health, disease and disability |  |  |  |  |  |  |  |  |  |  |
| 2. | Healthy nutrition as a precondition for building health potential |  |  |  |  |  |  |  |  |  |  |
| 3. | Physical activity and mobility as a source of health |  |  |  |  |  |  |  |  |  |  |
| 4. | Classification of factors of positive and negative effect on the human body |  |  |  |  |  |  |  |  |  |  |
| 5. | Pro-health, risky and anti-health behaviors |  |  |  |  |  |  |  |  |  |  |
| 6. | Creation of safe conditions for life, work, education, leisure and communication |  |  |  |  |  |  |  |  |  |  |
| 7. | Observation of functioning of own body – scope and importance of self-examination |  |  |  |  |  |  |  |  |  |  |
| 8. | Prophylactic examinations as an element of early detection of diseases |  |  |  |  |  |  |  |  |  |  |
| 9. | Preventive vaccination – myths and reality |  |  |  |  |  |  |  |  |  |  |
| 10. | Health situations acc. to need for medical consultation and self-treatment after consultation with a pharmacist |  |  |  |  |  |  |  |  |  |  |
| 11. | Provision of pre-medical care in emergency situations |  |  |  |  |  |  |  |  |  |  |

|  | | | | | | | | | | | |
| --- | --- | --- | --- | --- | --- | --- | --- | --- | --- | --- | --- |
|  | **Prevention of** | **1** | **2** | **3** | **4** | **5** | **6** | **7** | **8** | **9** | **10** |
| 12. | cardiovascular system diseases |  |  |  |  |  |  |  |  |  |  |
| 13. | neoplasms |  |  |  |  |  |  |  |  |  |  |
| 14. | psychiatric diseases |  |  |  |  |  |  |  |  |  |  |
| 15. | respiratory system diseases |  |  |  |  |  |  |  |  |  |  |
| 16. | genitourinary system diseases |  |  |  |  |  |  |  |  |  |  |
| 17. | spine and motor system diseases |  |  |  |  |  |  |  |  |  |  |
| 18. | stomatognathic system diseases |  |  |  |  |  |  |  |  |  |  |
| 19. | metabolic diseases |  |  |  |  |  |  |  |  |  |  |
| 20. | digestive system diseases |  |  |  |  |  |  |  |  |  |  |
|  | | | | | | | | | | | |

|  | **Copying with** | **1** | **2** | **3** | **4** | **5** | **6** | **7** | **8** | **9** | **10** |
| --- | --- | --- | --- | --- | --- | --- | --- | --- | --- | --- | --- |
| 21. | stress |  |  |  |  |  |  |  |  |  |  |
| 22. | pain |  |  |  |  |  |  |  |  |  |  |
| 23. | cold, infection and temperature fluctuations |  |  |  |  |  |  |  |  |  |  |
| 24. | digestive system disorders/symptoms and food poisoning |  |  |  |  |  |  |  |  |  |  |
| 25. | skin, mucous membrane and nail disorders |  |  |  |  |  |  |  |  |  |  |
| 26. | sleeping disorders |  |  |  |  |  |  |  |  |  |  |
| 27. | occupational health and safety |  |  |  |  |  |  |  |  |  |  |
| 28. | addictions |  |  |  |  |  |  |  |  |  |  |
| 29. | oedema |  |  |  |  |  |  |  |  |  |  |
| 30. | weight disorders |  |  |  |  |  |  |  |  |  |  |
| 31. | ocular and hearing problems |  |  |  |  |  |  |  |  |  |  |
| 32. | tinea infections |  |  |  |  |  |  |  |  |  |  |
| 33. | parasite infections |  |  |  |  |  |  |  |  |  |  |
| 34 | genitourinary tract infections |  |  |  |  |  |  |  |  |  |  |
| 35. | drug and other toxins poisoning |  |  |  |  |  |  |  |  |  |  |
| 36. | allergy |  |  |  |  |  |  |  |  |  |  |
