# Supplementary material for: Satisfaction of people at post-working age with pharmacists’ health promotion in Poland
Source: BMC Public Health. 2024 Jan 23;24:281. doi: 10.1186/s12889-024-17751-3 (PMC10807121; doi:10.1186/s12889-024-17751-3)
Supplement: Supplementary file 2 — Supplementary Material 2 [file 12889_2024_17751_MOESM2_ESM.docx]

**Health Behaviors Inventory**

Below there are examples of various health-related behaviors.

How often during the last year do you engage in the behaviors listed below?

Please answer honestly by entering the number representing your correct answer in each box:

1 – almost never; 2 – rarely; 3 – sometimes; 4 – often; 5 – almost always.

|  | **1** | **2** | **3** | **4** | **5** |
| --- | --- | --- | --- | --- | --- |
| 1. I eat a lot of vegetables and fruits |  |  |  |  |  |
| 1. I avoid colds |  |  |  |  |  |
| 1. I take seriously recommendations from those who express concern about my health |  |  |  |  |  |
| 1. I take sufficient rest |  |  |  |  |  |
| 1. I limit consumption of such products as animal fat and sugar |  |  |  |  |  |
| 1. I have written down (I know) the telephone numbers of ambulance services |  |  |  |  |  |
| 1. I avoid situations which have a depressing effect on me |  |  |  |  |  |
| 1. I avoid being overworked |  |  |  |  |  |
| 1. I care about correct nutrition |  |  |  |  |  |
| 1. I observe medical recommendations resulting from my examinations |  |  |  |  |  |
| 1. I try to avoid excessively strong emotions, stress and tensions |  |  |  |  |  |
| 1. I control my body weight |  |  |  |  |  |
| 1. I avoid consuming food containing preservatives |  |  |  |  |  |
| 1. I regularly report for health checkups |  |  |  |  |  |
| 1. I have friends and a regular family life |  |  |  |  |  |
| 1. I get sufficient sleep |  |  |  |  |  |
| 1. I avoid salt and heavily salted food |  |  |  |  |  |
| 1. I try to find out how others avoid diseases |  |  |  |  |  |
| 1. I avoid such feelings as anger, anxiety and depression |  |  |  |  |  |
| 1. I limit my tobacco consumption |  |  |  |  |  |
| 1. I eat wholegrain bakery products |  |  |  |  |  |
| 1. I try to obtain medical information and understand the causes of health and disease |  |  |  |  |  |
| 1. I think positively |  |  |  |  |  |
| 1. I avoid excessive physical effort |  |  |  |  |  |
